# Antecedents and Staff Outcomes Related to Multiple Dimensions of Perceived Person–Environment Fit Among Nurses: A Scoping Review

**DOI:** 10.1155/jonm/2914169

**Published:** 2026-08-11

**Authors:** Maho Inoue, Tamaki Isobe, Yoshie Takahashi, Kojiro Morita, Mari Ikeda

**Affiliations:** ^1^ Department of Nursing Administration, Division of Health Sciences and Nursing, Graduate School of Medicine, The University of Tokyo, Bunkyo, Tokyo, Japan, u-tokyo.ac.jp

## Abstract

**Aim(s):**

This study aimed to map the antecedents and outcomes related to each dimension of perceived person–environment fit (i.e., person–vocation, person–job, needs–supplies, demands–abilities, person–organization, person–supervisor, person–group fit, and value congruence) among nurses.

**Design:**

Scoping review.

**Methods:**

The population, concept, and context framework were adopted. Literature published up to August 14, 2024, was included. Four databases were searched: PubMed, Web of Science, CINAHL, and Ichushi‐Web. For each dimension of person–environment fit, we extracted antecedents and outcomes and then categorized conceptually similar variables within each set. The Preferred Reporting Items for Systematic reviews and Meta‐Analyses extension for Scoping Reviews (PRISMA‐ScR) was employed as the reporting method.

**Results:**

Forty‐five final sources were included after screening 1,385 articles. Thirty‐eight cross‐sectional studies were examined. No study examined person–vocation fit. Significant antecedents were predominantly categorized into individual and organizational factors, with most demographic factors showing no significant relationship. Outcomes were categorized as staff outcomes, including competence, work attitude, performance, perception of the workplace, and psychological states. Relationships with retention‐related factors, categorized under work attitude, varied in both the presence and direction across person–job and person–organization fit.

**Conclusion:**

It may be useful to actively sustain the specific dimensions of person–environment fit necessary for desired staff outcomes by leveraging both organizational support and nurses’ professional development behaviors.

**Implications:**

To enhance perceived person–environment fit, nurse managers should foster a supportive environment that encourages growth and learning behaviors. Moreover, this study identified a research gap concerning perceived person–environment fit among nurses, and it offers valuable insights for developing fit strategies for nurses. Future studies must explore perceived person–environment fit fluctuation patterns and person–vocation fit while also identifying distinct fit profiles through cluster analysis and examining potential curvilinear relationships between fit dimensions and staff outcomes. Furthermore, intervention studies, systematic reviews, and meta‐analyses could inform more effective fit strategies.

## 1. Introduction

The retention and recruitment of nurses are urgent global challenges owing to the increasing demand for nursing jobs [1]. It is thus crucial for nurses to be able to continue working with positive psychological attitudes, beneficial for both nurses’ occupational retention and improved well‐being. Diverse factors within the work environment, ranging from organizational support and compensation to job autonomy and team relationships, are associated with nurses’ retention and job satisfaction [2]; thus, it is important to focus on the interactions between the multifaceted work environment and individual nurses.

However, the environment surrounding nurses has been changing dynamically on multiple levels. Societal‐level changes, such as the COVID‐19 pandemic; facility‐level changes, such as organizational restructuring; and unit‐level changes, including changes in supervisors or colleagues, can sometimes threaten nurses’ career continuity [2, 3]. Additionally, approximately 70% of nurses transfer between facilities or departments [4], signifying potential opportunities too for them to work in diverse environments. However, environmental change could diminish employees’ sense of fit with the workplace [5]. If a sense of misfit persists, it could lead to negative psychological states and poor performance [6, 7]. Therefore, nurses require strategies to maintain a sense of fit with the multiple dimensions of their work environment, both when they proactively change the work environment and when it changes due to factors beyond their control.

This study focused on person–environment fit, defined as the compatibility between an individual and a work environment when their characteristics are well matched, representing the consequences of the interaction between the individual and work environment [8, 9]. However, since the rather simple initial definition of person–environment fit, emerging research has revealed its multidimensional nature [8]. Moreover, a diversification of measurement methods has been reported [10, 11].

First, the constituents of person–environment fit have multiple dimensions. For example, the work environment constitutes the vocation, job, organization, supervisor, and group, implying multiple types of fit: person–vocation, person–job, person–organization, person–supervisor, and person–group [8, 12, 13]. Additionally, the type of fit is classified according to how individual characteristics fit the work environment: needs–supplies fit (which occurs when the individual’s needs match those supplied by the workplace), demands–abilities fit (which occurs when the individual’s abilities match what is required by the work environment), and value congruence (i.e., the congruence between the individual’s values and those of their organization) [8, 12, 13]. Meta‐analyses of person–environment fit, mainly in the fields of management and organizational psychology, have reported that the presence and strength of relationships between person–environment fit and positive psychological and attitudinal outcomes differ for each dimension of the construct [8].

Second, the methods to measure person–environment fit are categorized into the following three types: (a) perceived fit, when an individual makes a direct assessment of the compatibility between themself and the environment, capturing their personal psychological assessment; (b) subjective fit, when fit is assessed indirectly by comparing a person’s report of their own characteristics with that of the environment’s characteristics; and (c) objective fit, when fit is assessed indirectly through a comparison using separate sources for the person and environment variables, aiming for a more neutral assessment [8]. Among these, a focus on strategies targeting perceived fit across diverse aspects of the work environment is likely to be effective for nurses’ occupational retention and well‐being because perceived fit is more strongly related to attitudinal variables than subjective or objective fit [8]. Notably, research on perceived person–environment fit targeting nurses has been increasing [14, 15]. Evidently, person–environment fit is a complex and multifaceted construct. Nevertheless, it would be beneficial to understand the outcomes and antecedents of each dimension of perceived person–environment fit, which could inform measures to help maintain and enhance each dimension among nurses, thus enabling them to work with a positive attitude.

Additionally, nurses are medical professionals engaged in work involving interpersonal interactions on a regular basis, and they have diverse work styles (i.e., employment and work patterns) and work at different types of institutions (e.g., hospitals, clinics, nursing homes, and home‐visit nursing services). Thus, unique factors related to perceived person–environment fit might exist among nurses. Moreover, the outcomes and antecedents may differ across dimensions of perceived person–environment fit because nurses experience fit across multiple aspects of the work environment. To gain insight into fit strategies that enable nurses to work while maintaining positive attitudes, as an initial step, it is necessary to comprehensively synthesize the knowledge regarding the antecedents and outcomes of the dimensions of perceived person–environment fit among nurses. A prior meta‐analysis has reported factors related to perceived person–organization, person–job, person–supervisor, person–group, needs–supplies, and demands–abilities fit, but the study did not focus on nurses [8]. Crucially, to our knowledge, no prior studies focusing on nurses have comprehensively synthesized the antecedents and outcomes of each dimension of perceived person–environment fit mentioned above.

## 2. Research Questions

The research questions addressed in this study were as follows.

1. What antecedents are related to high or low levels of each dimension of perceived person–environment fit (i.e., person–vocation, person–job, person–organization, person–supervisor, person–group, needs–supplies, demands–abilities fit, and value congruence) among nurses?
2. What outcomes are related to high or low levels of each dimension of perceived person–environment fit (i.e., person–vocation, person–job, person–organization, person–supervisor, person–group, needs–supplies, and demands–abilities fit, and value congruence) among nurses?

## 3. Aim

This study aimed to comprehensively map the following two aspects related to each dimension of perceived person–environment fit (i.e., person–vocation, person–job, needs–supplies, demands–abilities, person–organization, person–supervisor, person–group fit, and value congruence) among nurses:

1. Antecedents that are related to high or low levels of each dimension of perceived person–environment fit
2. Outcomes that are related to high or low levels of each dimension of perceived person–environment fit

## 4. Methods

### 4.1. Design

A scoping review was conducted to map the antecedents and outcomes across all major dimensions of perceived person–environment fit in the nursing population.

### 4.2. Key Definitions

The following concepts are the dimensions of person–environment fit directly assessed by individual nurses.

• Person–vocation fit: the compatibility between the nurse and the nursing profession/a career as a clinical nurse
• Person–job fit: the compatibility between the nurse and their current tasks/duties/roles that are performed at work
• Person–organization fit: the compatibility between the nurse and their organization
• Person–supervisor fit: the compatibility between the nurse and their supervisor
• Person–group fit: the compatibility between the nurse and their work group
• Needs–supplies fit: the compatibility that occurs when the nurse’s needs are met by their work environment (i.e., vocation, job, organization, supervisor, or group)
• Demands–abilities fit: the compatibility that occurs when the nurse’s skill meets the needs of their work environment (i.e., vocation, job, organization, supervisor, or group)
• Value congruence: the similarity between the nurse’s values and those of their organization, supervisor, or work group

### 4.3. Protocol and Registration

A protocol for this study was registered on the Open Science Framework (OSF) (OSF registration number (10.17605/OSF.IO/T8V37)). We used the Preferred Reporting Items for Systematic reviews and Meta‐Analyses extension for Scoping Reviews (PRISMA‐ScR) [16] to guide this review.

### 4.4. Eligibility Criteria

Eligibility criteria were set based on the population, concept, and context framework [17]. The population was registered nurses; the concept was perceived person–environment fit (i.e., person–vocation, person–organization, person–job, person–supervisor, person–group, needs–supplies, demands–abilities fit, and value congruence); and regarding the context, there were no restrictions on country, location, or social context.

#### 4.4.1. Inclusion Criteria

The inclusion criteria were as follows: (a) research examining registered nurses’ perceived person–environment fit (i.e., person–vocation, person–organization, person–job, person–supervisor, person–group, needs–supplies, demands–abilities fit, and value congruence that the nurses directly assessed); (b) research quantitatively examining the direct relationships between perceived person–environment fit and other concepts (i.e., quantitative or mixed‐methods studies); (c) original articles published in peer‐reviewed journals; and (d) articles written in English or Japanese. To maintain the scientific quality and rigor of the evidence, the gray literature, such as doctoral dissertations and conference abstracts, was excluded from this study.

#### 4.4.2. Exclusion Criteria

The exclusion criteria were as follows: (a) studies that focused on a different population (i.e., only reported analysis results for other professionals, nursing professionals other than registered nurses [e.g., advanced practice nurses, nurse assistants, and midwives], or nursing managers); (b) articles for which the full text was not accessible through open‐access or institutional library services; (c) research in which the measurement scale or method for assessing person–environment fit was unclear; (d) studies measuring objective fit (i.e., calculated by comparing scores of nurses and others) or subjective fit (i.e., calculated by researchers based on nurses’ independent evaluations of personal and environmental variables); and (e) articles with fundamental and unresolvable discrepancies between the data presented in their figures/tables and the text, as confirmed by unanimous consensus among all researchers. Regarding the exclusion criterion (a), to ensure a comprehensive collection of evidence related to registered nurses, studies that included other nursing professionals alongside registered nurses were retained. However, those studies focusing exclusively on advanced practice nurses, midwives, or nurse managers were excluded to maintain focus on the registered nurse, as their autonomy and roles were different from those of registered nurses.

### 4.5. Information Sources

On August 14, 2024, we searched the published literature from the inception of each database up to the search date using the following electronic bibliographic databases: PubMed, Web of Science, CINAHL, and Ichushi‐Web. No publication date restrictions were applied.

### 4.6. Search

The final search strategy for PubMed can be found in Supporting Information 1. To ensure a comprehensive literature search, librarians were consulted to provide feedback during the development of the search strategy. Specifically, they reviewed the choice of databases, search terms, and search strings in alignment with the study’s aim and research questions. The researchers and librarians collaboratively evaluated the preliminary results and iteratively refined the search terms and strings, in order to ensure that the search strategy was both appropriate and sufficiently sensitive.

### 4.7. Selection of Sources of Evidence

To increase consistency among reviewers, all researchers discussed and agreed on the screening process before beginning screening for this review. First, a researcher (MI) searched the literature following the information sources. Duplication was eliminated on the screening software Rayyan. Then, the remaining information sources were shared with all the researchers. Three researchers (MI, TI, and YT) independently evaluated whether the titles and abstracts met the inclusion criteria: MI evaluated all of the works, while TI and YT were responsible for evaluating each half. After discussion among all the researchers, the included articles to be read in full text were selected. Regarding full‐text availability, three researchers (MI, TI, and YT) independently evaluated whether the full text met the inclusion criteria: MI evaluated all of the works, while TI and YT were responsible for evaluating each half. Finally, all the researchers discussed disagreements on study selection and data extraction and reached a consensus on the final literature to be extracted.

### 4.8. Data Charting Process

Data were extracted by MI from the final literature. First, MI thoroughly read the full text of each article. Then, if a study included participants from other professions, nursing professionals other than nurses, or nurse managers, MI confirmed that only the findings pertaining to registered nurses were reported. Finally, the following data items were extracted and entered into an Excel file by MI. The research team reviewed the data items and discussed any ambiguities or their appropriateness. An agreement was ultimately reached among the research team that the data items were appropriate.

### 4.9. Data Items

The following data items were charted: the year of publication, country of study, study design, participants, examined dimensions of person–environment fit, measurement scales, analysis methods, and concepts related to person–environment fit.

Regarding the examined dimensions of person–environment fit, we noted that the terminology and measurement of person–environment fit were inconsistent across the literature during the screening process. For instance, some studies described assessing “subjective fit” but based on their measurement methods, we found that they in fact investigated “perceived person–organization fit” as defined in this review [18]. To account for this discrepancy, we recorded the dimension of person–environment fit as stated in each article under the data item “as stated in the literature.” Subsequently, one researcher (MI) reclassified each dimension based on the definitions used in this review, recording the result under “classification in this review.”

Additionally, we observed that among the included studies on person–job fit, research that utilized the work–life framework measured person–job fit in a distinct way. Specifically, in this framework, the areas of work–life are workload, control, reward, community, fairness, and values. In prior research, the person–job fit score was calculated either for each individual area or as a total score across all areas [19]. Therefore, under “classification in this review,” we added a category for “person–job fit/match (in the areas of work–life)” for studies that investigated person–job fit with this theoretical framework, in addition to the eight classifications presented in our definitions.

Finally, the related concepts with the dimensions of person–environment fit were categorized and recorded based on the type of analysis used to test their relationships. Concepts examined using noncausal analyses (e.g., correlation and *t*‐tests) were recorded under “factors not causally related to person–environment fit.” For concepts examined using causal analyses (e.g., multiple regression analysis and structural equation model), we created three categories. When person–environment fit was the dependent variable, we recorded work‐related independent variables under “work‐related antecedents of person–environment fit” and demographic independent variables under “demographic‐related antecedents of person–environment fit.” Conversely, when person–environment fit was the independent variable, concepts serving as dependent variables were recorded under “outcomes of person–environment fit.” The data were further distinguished by whether the study used a cross‐sectional or longitudinal design. Additionally, we recorded the direction of the relationship (i.e., positive or negative) and a nonsignificant relationship.

### 4.10. Quality Appraisal

As this study involved a scoping review, quality appraisal was not conducted because the objective was to map the breadth of available literature on a broad topic, offering an overview of the existing research regardless of its methodological quality. While a formal quality appraisal was not conducted, articles with fundamental data discrepancies were excluded to ensure synthesis reliability (see Exclusion Criteria [e]).

### 4.11. Synthesis of Results

“Work‐related antecedents of person–environment fit,” “demographic‐related antecedents of person–environment fit,” and “outcomes of person–environment fit” were mapped. Specifically, we integrated the work‐related and individual‐related antecedents into a single category labeled “antecedents,” and the outcomes were labeled “outcomes.” We then created a table for each dimension of person–environment fit, differentiating between concepts with positive, negative, and nonsignificant relationships. Concepts from cross‐sectional studies were recorded under “measured at the same time as person–environment fit,” while concepts from longitudinal studies were recorded under “measured before/after person–environment fit.”

Subsequently, we categorized similar mapped concepts by grouping them and denoting the category names.

## 5. Results

### 5.1. Selection of Sources of Evidence

**FIGURE 1:**
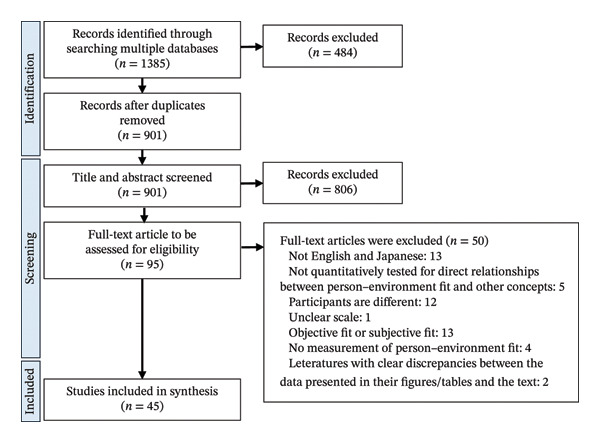
Selection of sources of evidence.

Figure 1 presents a flowchart depicting the process from the literature search based on the final strategy to the selection of sources of evidence. First, 1,385 articles were searched from four databases. Subsequently, 484 duplications were excluded, and a two‐step screening process was conducted. The final dataset included 45 sources.

### 5.2. Characteristics of Sources of Evidence

**FIGURE 2:**
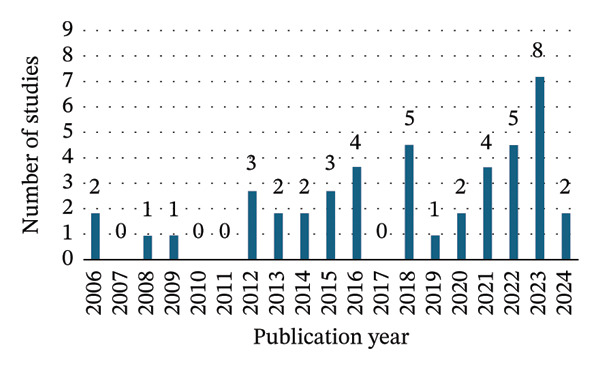
Publication year of the included studies (*n* = 45).

The first publication year of the included literature was 2006, and the number of published papers gradually increased from the 2010s (Figure 2). Regarding the examined dimensions of person–environment fit, there were cases where multiple dimensions were measured in a single study. The examined dimensions of person–environment fit, as categorized in this review, were counted as follows: person–vocation fit (*n* = 0), person–job fit (*n* = 13), person–organization fit (*n* = 21), person–supervisor fit (*n* = 4), person–group fit (*n* = 6), needs–supplies fit (*n* = 5), demands–abilities fit (*n* = 5), value congruence (*n* = 10), and person–job fit/match (in the areas of work–life) (*n* = 6) (Table 1).

**TABLE 1:** Examined dimensions of person–environment fit.

Classification in this review	Number of studies
Person–vocation fit	0
Person–job fit	13
Person–organization fit	21
Person–supervisor fit	5
Person–group fit	6
Needs–supplies fit	5
Demands–abilities fit	5
Value congruence	10
Person–job fit/match (in the areas of work–life)	6

Many scales were used for measuring each dimension of perceived person–environment fit (Table 2). Even when the same scale was used, there were cases where the number of items and the response method (e.g., Likert scale) differed across studies. Furthermore, even when the reported dimension of person–environment fit was the same across studies, the measured concept often differed because the scale used was different. For example, although both the perceived fit scale [10] and the perceived person–environment fit scale [11] are commonly used, they measure distinct domains of person–organization fit. The former measures the congruence of individual and organizational values, while the latter measures the congruence of individual and organizational values and goals.

**TABLE 2:** Measurements of person–environment fit.

References cited in the final examined literature for the scales or items	Classification in this review	Number of studies
Cable and DeRue (2002) [10]	Person–job fit	4
	Needs–supplies fit	1
	Demands–abilities fit	1
	Person–organization fit	6
	Value congruence	1

Cable and DeRue (2002); Inoue et al. (2021) [10, 20]	Needs–supplies fit	4
	Demands–abilities fit	4
	Person–organization fit	4
	Person–supervisor fit	3
	Person–group fit	4

Cable and Judge (1996) [21]	Person–organization fit	3

Chuang et al. (2016) [11]	Person–job fit	4
	Person–organization fit	2
	Person–supervisor fit	2
	Person–group fit	2

Kristof (1996) [13]	Person–organization fit	1

Lauver and Kristof‐Brown (2001) [22]	Person–job fit	2

Leiter and Maslach (2000) [23]	Person–job fit/match (in the areas of work–life)	1

Leiter and Maslach (2004) [24]	Person–job fit/match (in the areas of work–life)	2
	Match in values (in the areas of work–life)	1
	Value congruence	1

Leiter and Maslach (2006) [25]	Value congruence	1

Leiter and Maslach (2011) [26]	Person–job fit/match (in the areas of work–life)	1

Maslach and Leiter (1997) [27]	Person–job fit/match (in the areas of work–life)	2

Netemeyer et al. (1997) [28]	Person–organization fit	2

Posner (1992); Jung and Yoon (2012) [29, 30]	Value congruence	1

Resick et al. (2007) [31]	Person–organization fit	1

Saks and Ashforth (1997) [32]	Person–job fit	2

Saks and Ashforth (2002) [33]	Person–job fit	1
	Person–organization fit	1

Supelli and Creed (2014) [34]	Person–organization fit	1

Xiang and Xiong (2018) [35]	Person–organization fit	1

Original items developed in the current study	Value congruence	4

Among the 45 studies examined, many were conducted in Canada (*n* = 8), China (*n* = 7), and the United States (*n* = 6), and the others were distributed across Asia, Europe, Africa, and Oceania. A full list of countries is provided in Supporting Information 2.

The number of cross‐sectional and longitudinal studies was 38 and 7, respectively.

### 5.3. Results of Individual Sources of Evidence

Supporting Information 3 summarizes the studies included in the synthesis.

### 5.4. Synthesis of Results

**FIGURE 3:**
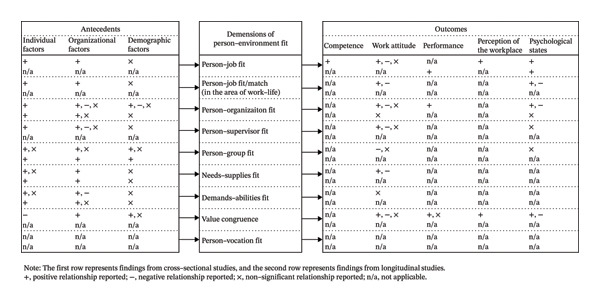
Map of the antecedents and outcomes of perceived person–environment fit among nurses.

Antecedents and outcomes of perceived person–environment fit among nurses were mapped (Table 3 and Figure 3). The number of reported concepts varied across the different dimensions of person–environment fit. Some concepts listed under “factors not causally related to person–environment fit” (Supporting Information 3) had not been tested for a relationship using a causal analysis. Furthermore, many antecedents and outcomes were investigated concurrently with person–environment fit in cross‐sectional studies.

**TABLE 3:** Antecedents and outcomes of perceived person–environment fit among nurses.

Dimensions of PE fit	Antecedents						Outcomes					
	Measured at the same time as PE fit			Measured before PE fit			Measured at the same time as PE fit			Measured after PE fit		
	+	−	×	+	−	×	+	−	×	+	−	×
Person–vocation fit	n/a	n/a	n/a	n/a	n/a	n/a	n/a	n/a	n/a	n/a	n/a	n/a

Person–job fit	# Individual factors Digital competence Self‐efficacy # Organizational factors Work–life balance practices	n/a	# Demographic factors Age Sex Working years	n/a	n/a	n/a	# Competence Cultural competence Work–life balance # Work attitude Job satisfaction Organizational identification Possibility of access to external work Retention Work engagement # Perception of the workplace Psychological climate	# Work attitude Difficulty in adapting Motivation in finding other employment Organizational cynicism Possibility of quitting the present job (‐/×) Turnover intention	# Psychological states Anxiety Depression #Work attitude Employee–customer identification Intent to leave job or organization	# Performance Job performance Organizational citizenship behavior # Psychological states Workplace wellbeing	n/a	n/a

Person–job fit/match (in the areas of work−life)	# Organizational factors Authentic leadership Structural empowerment Empowerment^a^ # Individual factors Psychological capital	n/a	n/a	n/a	n/a	n/a	# Psychological states Occupational coping self‐efficacy Emotional exhaustion^b^ # Work attitude Civility norms Person−job fit/match in Community^c^ Person–job fit/match in Fairness^c^ Person–job fit/match in Reward^c^ Person–job fit/match in Workload^c^	# Work attitude Bullying experiences # Psychological states Burnout Emotional exhaustion	n/a	n/a	n/a	n/a

Person–organization fit	# Organizational factors Colleagues′ support: • Appraisal support • Emotional support • Information support Emotional intelligence Flexibility‐promoting change Leader–member exchange Nurse managers’ support: • Appraisal support • Emotional support • Information support • Instrumental support On‐the‐job training: • Consultation • Teaching Organizational identification Perceived reflection support from: • Colleagues • Nurse managers Transformational leadership Workplace spirituality # Demographic factors Hospital (+/×) Total years of nursing experience	# Organizational factors Flexibility‐limiting change # Demographic factors (‐/×) Education (‐/×) Sex	# Organizational factors Assignment to the nurses’ department of choice Colleagues′ support: • Instrumental support # Individual factors Individual learning behaviors: • Learning from practice • Learning from training • Learning through reflection Operational changes # Demographic factors Age Employment position Experience Number of prior affiliated facilities Nursing unit Qualification Recently hired with prior clinical experience Total years in the current nursing unit	# Individual factors; previous fit Person–organization fit # Organizational factors On‐the‐job opportunities for professional growth (+/×) Communication about the nursing practice with colleagues (+/×) Learning in hospitals (+/×) Learning outside hospitals	n/a	# Demographic factors Employment position Nursing unit Sex Total years in the current nursing unit Total years of nursing experience	# Work attitude Organizational commitment Organizational identification Organizational loyalty Work engagement (+/×) Job satisfaction # Psychological states Psychological empowerment # Performance Doctor‐rated innovative work behavior Innovative behavior Innovative work behavior (average of self‐rated and doctor‐rated) Knowledge‐sharing behavior Self‐rated innovative work behavior	# Psychological states Administrative stressors Anxiety Depression # Work attitude Motivation in finding other employment Possibility of access to external work Possibility of quitting the present job Turnover intention	# Work attitude Employee–customer identification Perceived job stress	n/a	n/a	# Work attitude Intention to leave the organization Intention to leave the profession # Psychological states Exhaustion means between Times 1 and 2 Psychological well‐being

Person–supervisor fit	# Organizational factors Colleagues′ support: • Appraisal support • Emotional support • Information support • Instrumental support Nurse managers’ support: • Appraisal support • Emotional support • Information support • Instrumental support On‐the‐job training: • Consultation • Teaching Perceived reflection support from: • Nurse managers # Individual factors Individual learning behaviors: • Learning from feedback • Learning from others • Learning from practice • Learning through reflection	# Organizational factors Operational changes	# Organizational factors Assignment to the nurses’ department of choice Individual learning behaviors: • Learning from training Perceived reflection support from: • Colleagues # Demographic factors Employment position Hospital Number of prior affiliated facilities Nursing unit Qualification Recently hired with prior clinical experience Sex Total years in the current nursing unit Total years of nursing# experience	n/a	n/a	n/a	# Work attitude Job satisfaction	# Work attitude Possibility of quitting the present job	# Work attitude Motivation in finding other employment Possibility of access to external work Turnover intention # Psychological state Depression	n/a	n/a	n/a

Person–group fit	# Organizational factors Colleagues′ support: • Appraisal support • Emotional support • Information support • Instrumental support Nurse managers’ support: • Appraisal support • Emotional support • Information support • Instrumental support On‐the‐job training: • Consultation • Teaching Perceived reflection support from: • Colleagues • Nurse managers # Individual factors Individual learning behaviors: • Learning from feedback • Learning from others • Learning from practice	# Demographic factors Nursing unit	# Organizational factors Assignment to the nurses’ department of choice Operational changes # Individual factors Individual learning behaviors: • Learning from reflection • Learning from training # Demographic factors Employment position Hospital Number of prior affiliated facilities Qualification Recently hired with prior clinical experience Sex Total years in current nursing unit Total years of nursing experience	# Individual factors; previous fit Person–group fit # Organizational factors On‐the‐job opportunities for professional growth (+/×) Learning in hospitals (+/×) Learning outside hospitals (+/×) Communication about the nursing practice with colleagues	n/a	# Demographic factors Employment position Nursing unit Sex Total years in current nursing unit Total years of nursing experience	n/a	# Work attitude (‐/×) Turnover intention	# Work attitude Motivation in finding other employment Possibility of access to external work Possibility of quitting the present job	n/a	n/a	n/a

Needs–supplies fit	# Organizational factors Assignment to the nurses’ department of choice; as requested Colleagues′ support: • Appraisal support • Emotional support • Information support • Instrumental support Nurse managers’ support: • Appraisal support • Emotional support • Information support • Instrumental support On‐the‐job training: • Consultation • Teaching Perceived reflection support from: • Colleagues • Nurse managers #Individual factors Individual learning behaviors: • Learning from feedback • Learning from practice	n/a	# Individual factors Individual learning behaviors: • Learning from others • Learning from training • Learning through reflection Operational changes # Demographic factors Employment position Hospital Number of prior affiliated facilities Nursing unit Qualification Recently hired with prior clinical experience Sex Total years in current nursing unit Total years of nursing experience	# Individual factors; previous fit Needs–supplies fit # Organizational factors On‐the‐job opportunities for professional growth (+/×) Learning in hospitals (+/×) Learning outside hospitals (+/×) Communication about the nursing practice with colleagues	n/a	# Demographic factors Employment position Nursing unit Sex Total years in the current nursing unit Total years of nursing experience	# Work attitude Work engagement	# Work attitude Turnover intention	n/a	n/a	n/a	n/a

Demands–abilities fit	# Organizational factors Assignment to the nurses’ department of choice; as requested Colleagues′ support: • Appraisal support • Emotional support • Information support • Instrumental support Nurse managers’ support: • Appraisal support • Emotional support • Information support • Instrumental support On‐the‐job training: • Consultation • Teaching Perceived reflection support from: • Colleagues • Nurse managers # Individual factors Individual learning behaviors: • Learning from feedback • Learning from others • Learning from practice • Learning through reflection	# Organizational factors Operational changes	# Individual factors Individual learning behaviors: • Learning from training # Demographic factors Employment position Hospital Nursing unit Number of prior affiliated facilities Nursing unit Qualification Recently hired with prior clinical experience Sex Total years in current nursing unit Total years of nursing experience	# Individual factors; previous fit Demands–abilities fit # Organizational factors On‐the‐job opportunities for professional growth (+/×) Communication about the nursing practice with colleagues (+/×) Learning in hospitals (+/×) Learning outside hospitals	n/a	# Demographic factors Employment position Nursing unit Sex Total years in the current nursing unit Total years of nursing experience	n/a	n/a	# Work attitude Turnover intention	n/a	n/a	n/a

*Note:* PE fit, person–environment fit; +, positive relationship; −, negative relationship; ×, nonsignificant relationship; #, category name; n/a, not applicable; (+/×), both positive and nonsignificant relationships were reported; (−/×), both negative and nonsignificant relationships were reported.

^a^The variable was the antecedent of “person–job fit/match vis‐à‐vis workload, control, rewards, community, fairness, and values.”

^b^The variable was the outcome of “person–job fit/match vis‐à‐vis workload.”

^c^These variables were the outcomes of “person–job fit/match vis‐à‐vis control.”

Overall, the most frequently reported significant antecedents for the various dimensions of person–environment fit were individual factors and organizational factors. Most of the demographic factors examined showed no significant relationship. Meanwhile, the outcomes investigated were competence, work attitude, performance, perception of the workplace, and psychological states. These relationships varied depending on the specific concepts and the examined dimensions of person–environment fit.

#### 5.4.1. Person–Vocation Fit

No study examining person–vocation fit was included in the final sources.

#### 5.4.2. Person–Job Fit

Only a few significant factors assessed as antecedents were reported for person–job fit, all of which showed a positive relationship: digital competence, self‐efficacy [67], and work–life balance practices [61].

As for factors assessed as outcomes, concepts categorized under competence and performance demonstrated a positive relationship. Work attitude showed both positive and negative relationships depending on the specific concepts measured. Notably, for concepts related to retention, retention had a positive relationship [40], and motivation in finding other employment and the possibility of quitting the present job had a negative relationship [41]. Conversely, the possibility of access to external work showed a positive relationship [41]. No significant relationship was reported with the intent to leave a job or organization [71]. Furthermore, studies reported a significant positive relationship between person–job fit and job performance, organizational citizenship behavior [53], and workplace well‐being [67], even when measured during a period of time after fit was assessed.

#### 5.4.3. Person–Job Fit/Match (in the Areas of Work–Life)

In cross‐sectional studies, factors assessed as antecedents included a positive relationship with organizational factors such as authentic leadership [63, 64] and empowerment [19, 57], as well as with the individual factor of psychological capital [62].

Factors assessed as outcomes showed a positive relationship with positive psychological states (e.g., occupational coping and self‐efficacy) [63] and a negative relationship with negative psychological states (e.g., burnout) [51, 62]. A distinctive finding was that a good fit with one area of work–life, control, was positively associated with fit in other areas: community, fairness, reward, and workload [19].

#### 5.4.4. Person–Organization Fit

A distinctive finding for potential antecedents was the positive relationships with organizational factors such as nurse managers’ and colleagues’ support [38, 60], on‐the‐job training [38], leadership characteristics (e.g., transformational leadership) [69], and workplace characteristics (e.g., flexibility‐promoting change and workplace spirituality) [18, 43]. Additionally, some studies reported differences based on demographic factors such as affiliated hospital, total years of nursing experience, educational background, and sex [60, 69]. In terms of longitudinal relationships, a previous level of person–organization fit and on‐the‐job opportunities for professional growth showed positive relationships [14].

Regarding potential outcomes measured concurrently with person–organization fit, a notable finding was the positive relationships with concepts representing the connection between the individual and organization (e.g., organizational commitment, identification, and loyalty) [15, 18, 49, 76], as well as with concepts representing performance rated by nurses themselves and doctors [47]. Furthermore, regarding psychological states, a positive relationship was found with positive concepts (e.g., psychological empowerment) [42], while negative relationships were found with negative concepts (e.g., administrative stressor) [18]. Although some studies investigated the longitudinal relationship between person–organization fit and work attitudes and psychological states, no significant relationships were reported [45, 77].

#### 5.4.5. Person–Supervisor Fit

Only cross‐sectional studies were reported. Regarding factors assessed as antecedents, positive relationships were found with organizational factors such as colleagues’ and nurse managers’ support and on‐the‐job training [38, 60], as well as individual factors such as individual learning behaviors [38]. Organizational change showed a negative relationship [60].

For factors assessed as outcomes, regarding retention‐related concepts, a negative relationship was found with the possibility of quitting the present job, but there were no significant relationships with motivation in finding other employment, the possibility of access to external work, or turnover intention [41].

#### 5.4.6. Person–Group Fit

For factors assessed as antecedents, organizational factors, such as colleagues’ and nurse managers’ support and on‐the‐job training [38, 60], and individual factors, such as individual learning behaviors, were positively related [38]. One study reported differences based on the nursing unit as a demographic variable [60]. Longitudinal relationships mirrored those found for person–organization fit [14].

As for potential outcomes, some studies reported a negative relationship with turnover intention [52], while others reported no significant relationship [41].

#### 5.4.7. Needs–Supplies Fit

Regarding factors assessed as antecedents, measured concurrently with needs–supplies fit, organizational factors such as assignment to the department that nurses requested, colleagues’ and nurse managers’ support, and on‐the‐job training were included [38, 60]. Individual factors included two elements of individual learning behaviors [38]. However, three elements of individual learning behaviors revealed no significant relationships [38]. Longitudinal relationships mirrored those found for person–organization fit [14].

Regarding factors assessed as outcomes, only a positive relationship with work engagement and a negative relationship with turnover intention were reported [72].

#### 5.4.8. Demands–Abilities Fit

The results for potential antecedents were similar to those for needs–supplies fit, but in this case, four elements of individual learning behaviors had a positive relationship [38]. Organizational change revealed a negative relationship [14, 60].

As for factors assessed as outcomes, only a nonsignificant relationship with turnover intention was reported [72].

#### 5.4.9. Value Congruence

Only cross‐sectional studies were reported. Positive relationships were reported with fairness and leader characteristics as organizational factors [65, 68]. A noteworthy finding was the positive relationship with the demographic variable of generation. Meanwhile, there was a negative relationship with self‐emotional intelligence [68].

Some relationships with work attitude were reported as outcomes. Regarding retention‐related concepts, a positive relationship was found with behavioral intentions to leave the nursing profession, while a negative relationship was reported with behavioral intentions to leave a current job [55]. Negative relationships were also reported for negative psychological states [65, 75]. Regarding performance, patient care quality had a positive relationship [74], while organizational citizenship behavior revealed a nonsignificant one [68].

## 6. Discussion

### 6.1. Overview and Study Novelty

To our knowledge, this study is the first comprehensive scoping review to map the antecedents and outcomes across all major dimensions of perceived person–environment fit in the nursing population. While previous reviews have reported on staff outcomes related to some dimensions of person–environment fit [8, 36], the comprehensive mapping of factors in this study, especially antecedents, offers a valuable contribution to person–environment fit research. However, in the body of research on nurses’ perceived person–environment fit, cross‐sectional studies significantly outnumbered longitudinal ones. Although this review categorizes both antecedents and outcomes separately for cross‐sectional and longitudinal designs (Table 3), the findings, especially those from cross‐sectional studies, do not provide robust evidence of causality regarding perceived person–environment fit.

Regarding the potential antecedents that could enhance the various dimensions of perceived person–environment fit, the overall trend indicated that significant relationships primarily fell into the organizational and individual factors categories, often including factors related to the evaluation of and reaction to practice. Most demographic variables showed no significant relationships. Since nursing is a specialized profession requiring expert knowledge and skills, this review notably identified factors contributing to high‐quality nursing practice—such as informational and instrumental support from nurse managers and colleagues, learning through practice, and on‐the‐job training—as well as psychological resources such as self‐efficacy, as characteristic factors [38, 67]. Furthermore, the nursing profession is marked by diverse work patterns, including shift work, and previous research has established work–life balance as a relative factor of retention [2]. It is particularly distinctive that work–life balance practices were reported as factors specifically associated with person–job fit in this context [61]. This is further evidenced by the inclusion of multiple studies that utilized the concept of person–job fit or match, specifically within the “areas of work‐life,” thereby reflecting an integrated approach that encompasses both professional and personal perspectives [19, 62–64].

Potential outcomes of perceived person–environment fit were largely staff outcomes—reporting on nurses’ work attitudes, psychological states, perceptions of the workplace, and performance—with no patient outcomes reported. Patient outcomes serve as essential performance indicators in nursing. Nevertheless, the pathway from perceived person–environment fit—a psychological indicator reflecting the interaction between an individual nurse and their work environment—to patient outcomes is likely obscured by the inherent complexities of interdisciplinary team‐based interventions. This complexity may explain why such relationships have remained largely unexplored to date. Conversely, the consistent reporting of staff outcomes, which reflect individual behaviors and attitudes, demonstrates that research grounded in person–environment fit theory [8, 9] has been actively conducted. This suggests that a substantial and valuable body of knowledge is being accumulated, particularly from the human resource management perspective.

### 6.2. Trend of Perceived Person–Environment Fit Research Among Nurses

There was a clear, uneven distribution in the person–environment fit dimensions investigated in the studies. The most frequently studied dimensions were person–organization and person–job fit. These two dimensions were often examined in relation to staff outcomes such as work attitude (e.g., retention, turnover intention, job satisfaction, and organizational commitment) [15, 18, 40, 41, 43, 44, 49, 50, 52, 56, 61, 71, 72, 76], performance (e.g., innovative behavior) [42, 47, 53, 79], and psychological states (e.g., burnout, anxiety, and depression) [42, 45, 67, 77]. This prominence likely reflects the critical organizational interest in nurse retention, the demand for high professional skills, and the emotional labor inherent in the profession, leading researchers to prioritize fit with the organization and current job.

While the diverse dimensions of person–environment fit are increasingly focused on in research, this review further highlights the crucial importance of distinguishing between these dimensions. A meta‐analysis targeting other professions has reported that the presence or absence of a relationship with a specific outcome may vary across person–environment fit dimensions [8], but it did not demonstrate a reversal in the direction of the relationship. Similarly, this scoping review confirmed that the existence of a relationship could vary by person–environment fit dimension. Crucially, however, the current findings revealed that while most person–environment fit dimensions have positive relationships with nurses’ retention intentions [40, 41, 44, 52, 72], some research suggests that a high degree of person–job fit and value congruence could, conversely, be related to high intention to leave nursing profession or access to external work [41, 55]. This finding further supports the imperative to distinguish and address the specific elements that fit particular aspects of the work environment. Ultimately, the inconsistencies found underscore the need for further research.

### 6.3. Antecedents to Increase Nurses’ Perceived Fit

In all dimensions of person–environment fit, significant positive factors assessed as antecedents included both organizational and individual ones. Given that these findings are predominantly based on cross‐sectional data, nurses with a strong sense of fit could possibly tend to report higher levels of organizational and individual factors. Nevertheless, this outcome suggests that even when a nurse perceives a misfit with the work environment, their sense of fit can be enhanced through organizational interventions and the nurse’s own cognition and behavior. The finding that organizational factors act as antecedents aligns with career development theory [37], which posits that organizational management practices—such as job assignment, training, and development—are essential for harmonizing individual and organizational goals. This review could serve as a valuable resource for future nursing management practice and research by providing an overview of diverse organizational factors that can enhance perceived person–environment fit, including nurse manager support, peer support, on‐the‐job training, leadership characteristics, and opportunities for professional growth.

Another noteworthy finding is that individual factors serve as antecedents, considering the theory of person–environment interaction [8, 9]. Notably, reporting individual learning behaviors as an individual factor [38] is important because the sense of fit can be enhanced through individual effort. Multiple elements of individual learning behaviors showed significant positive relationships with needs–supplies, demands–abilities, person–supervisor, and person–group fit [38]. The International Council of Nurses [39] mandates that nurses engage in continuous professional development as a professional responsibility. Therefore, it is suggested that by proactively maintaining this commitment and engaging in learning behaviors, nurses not only fulfill their professional duty but may also enhance their sense of fit with multiple aspects of the work environment. This, in turn, could enable them to adopt positive work attitudes and mitigate negative psychological states.

However, individual learning behaviors had a nonsignificant relationship with person–organization fit [38]. The significant factors assessed as antecedents reported for person–organization fit were predominantly organizational in this review. It appears that person–organization fit is primarily fostered by opportunities to understand the organization’s values and policies through organizational support, leadership, and the workplace climate. As individual learning behaviors are often focused on in daily clinical practice, they might not relate to person–organization fit.

### 6.4. Contrasting Roles of Fit in Nurse Retention

Regarding potential outcomes, there was no consistent consensus across the dimensions of person–environment fit regarding their relationship with retention or turnover‐related concepts, as reported previously. Person–job fit was reported to have a positive relationship with retention [40]. However, another study [41] reported a negative relationship with motivation in finding other employment and the possibility of quitting the present job, alongside a positive relationship with the possibility of access to external work, and no significant relationship with turnover intention. These may be unique findings for nurses in comparison with the results of a prior meta‐analysis in the management and organizational psychology fields, which did not focus on nurses [8]. A high degree of person–job fit may not guarantee organizational retention, potentially because nurses who feel well‐suited to their current role could also gain confidence from the fact that their skills are transferable to other organizations. In the future, examining the curvilinear relationship between person–job fit and retention/turnover could provide new insights.

Conversely, cross‐sectional studies on person–organization fit consistently reported negative relationships with motivation in finding other employment, the possibility of access to external work, the possibility of quitting the present job, and turnover intention [42–44], with no counterevidence reported. This finding suggests that nurses with a high degree of person–organization fit have a strong intention to stay with the specific organization they are working with. Nevertheless, a longitudinal study found that even person–organization fit had no significant relationship with the intention to leave the organization or intention to leave the profession 1 year later [45]. Therefore, to promote nurse retention within an organization, it is essential to ensure that a high sense of fit with the organization is sustained over time. However, if the goal is broader—such as retaining nurses in the general labor market or prioritizing competence and performance improvement, supporting a high degree of person–job fit is equally crucial.

However, it should be noted that this review did not identify any studies that measured actual turnover as an outcome. Since the included studies quantified turnover intention or the likelihood of leaving to assess their relationship with person–environment fit [41–45], the direct relationship with actual turnover remains unexamined. Therefore, further investigation into each dimension of person–environment fit and actual turnover is necessary.

### 6.5. Implications for Strategies Focused on Person–Environment Fit

#### 6.5.1. Theoretical Implications and Future Research

To advance the field of person–environment fit in nursing, future research should focus on the following three categories.

##### 6.5.1.1. Conceptual Clarification

First, the results suggest that a thorough understanding of the various person–environment fit concepts and their measurement scales is essential for person–environment fit research. The diversity in person–environment fit dimensions naturally leads to a diversity in measurement scales. Researchers must therefore carefully consider which concept is being measured when comparing or applying the findings from previous studies.

##### 6.5.1.2. Focus on Person–Vocation Fit

Second, no research on person–vocation fit among nurses was found in the final literature examined. As the construct of person–vocation fit is rooted in vocational development and choice theories [8, 13], nursing administrators within healthcare organizations may have historically prioritized enhancing fit within the organization over fit with the profession. However, previous research focusing on resident physicians suggests that a high level of person–vocation fit could prevent burnout [46]. If the goal is to enable nurses to sustain the nursing profession by adapting to environmental changes, it may be vital to support the maintenance and enhancement of vocational fit. Person–vocation fit is similar to person–job fit but is considered the broadest level of work–environment fit [13]. Future research investigating antecedents and outcomes of perceived person–vocation fit, while clearly differentiating it from other dimensions, could provide valuable new knowledge. Achieving this requires the development of a reliable and valid perceived person–vocation fit scale specifically tailored for the nursing context.

##### 6.5.1.3. Methodologically Refined Investigation of Antecedents and Outcomes

Third, it must be noted that person–environment fit is a dynamic concept. The literature examined in this review predominantly encompassed cross‐sectional observational research. A previous study has indicated that the temporal pattern of perceived value congruence is dynamic, indicating *dynamic*, *misfit*, and *stable* trends [7]. As the person–environment fit concept is rooted in interactional psychology [8], it could evolve based on shifts in individual needs, abilities, or values, as well as perceptions of the workplace environment. Therefore, in future research, it is critical to explicitly hypothesize the temporal variability of person–environment fit. Given that multiple concepts showed relationships in noncausal analyses (Supporting Information 3), although not featured in Table 3, future research should involve prospective longitudinal studies to verify the following three aspects: the fluctuation patterns of each person–environment fit dimension, their relationships with antecedents, and their long‐term impact on staff outcomes. Furthermore, conducting intervention studies, systematic review, and meta‐analyses will be essential to establish robust evidence regarding the causal relationships between the factors identified in this review and person–environment fit.

Lastly, future research should explore the underlying mechanisms of staff outcomes through more sophisticated analytical approaches. These include identifying distinct profiles through cluster analysis based on scores across various person–environment fit dimensions (e.g., groups with high person‐job but low person–organization fit), as well as examining potential curvilinear relationships between specific fit dimensions and staff outcomes. Such approaches will provide a more nuanced understanding of how nursing retention and well‐being are generated.

#### 6.5.2. Practical Implications

This study contributes to knowledge on the dimensions of person–environment fit that are desirable for achieving specific staff outcomes. Significantly, outcomes vary by dimension, and a high sense of fit with various aspects of the work environment may lead to positive attitudes, psychological states, and workplace perceptions, as well as high performance in nurses. Assessing the level of nurses’ perceived person–environment fit can help nurse managers confirm or predict whether anticipated staff outcomes are likely to occur. Therefore, it might be useful for nurse managers to understand the specific dimensions of fit that nurses do or do not report. Nurse managers could strategically implement the reported antecedents of a specific dimension of nurses’ person–environment fit―for instance, by fostering a supportive environment that encourages growth―when aiming to enhance outcomes related to the particular dimension.

The study also suggests that nurses themselves have the potential to enhance their perceived fit with the work environment through their own actions. Perceived person–environment fit can be intentionally changed through nurses’ learning behavior [38]. Therefore, by raising self‐awareness of their multidimensional person–environment fit levels and engaging in learning behaviors, nurses may foster positive work attitudes, psychological well‐being, and performance, ultimately leading to a sustainable career. That is, when nurses experience a misfit in the workplace and seek to improve the specific dimension of person–environment fit, they would benefit from actively engaging in the learning behaviors reported as significant antecedents of that dimension. Additionally, nurse managers should create an environment that encourages nurses to engage in these learning behaviors.

### 6.6. Limitations

This study involved a scoping review, which allowed us to map the various dimensions of perceived person–environment fit and their related factors among nurses. However, even within the same dimension of person–environment fit, some related factors showed inconsistent findings, such as the relationship between person–job fit and retention/turnover. The possibility that the examined literature included studies with designs of lower quality cannot be ruled out. Furthermore, the elements being measured may have varied across studies. Therefore, to raise the level of evidence regarding the relationships between person–environment fit dimensions and their related factors, future research must involve systematic reviews or meta‐analyses that assess study quality and focus on specific measured elements or measurement scales.

Moreover, because the included literature was limited to articles published in English and Japanese, this review does not capture evidence reported in papers published in other languages. Consequently, there may be additional factors related to nurses’ perceived person–environment fit beyond those reported here. Indeed, unexamined studies reported in articles published in other languages may show differing results regarding the presence or direction of relationships for the antecedents and outcomes identified in this review.

Furthermore, a limitation arises regarding the temporal scope and reproducibility of the literature search, which was conducted on August 14, 2024. Since most academic databases do not support filtering by specific months and categorize publications by year, a replication of this search may include articles published until December 31, 2024. Consequently, this systemic constraint inherently affects the precise reproducibility of the search results for 2024.

As this study primarily focused on registered nurses, the findings may have limited generalizability to nursing professionals other than registered nurses (e.g., advanced practice nurses and midwives), or nursing managers, whose professional frameworks and autonomy may influence the antecedents and outcomes of person–environment fit differently.

## 7. Conclusion

This scoping review mapped the antecedents and outcomes of eight dimensions of perceived person–environment fit (i.e., person–vocation, person–job, person–organization, person–supervisor, person–group, needs–supplies, demands–abilities fit, and value congruence) and person–job fit/match (in the areas of work–life) among nurses. Studies focusing on person–organization fit were the most in number. Meanwhile, no research on person–vocation fit among nurses was found. Antecedents were primarily categorized as individual factors and organizational factors, with most demographic factors showing no significant relationship. Outcomes included competence, work attitude, performance, perception of the workplace, and psychological states, with relationships varying across the different dimensions of fit.

This study suggests that fit strategies for nurse retention and well‐being should move beyond a simple view of person–environment fit. It could be useful to actively sustain the specific dimensions of person–environment fit necessary for desired staff outcomes by leveraging both organizational support and nurses’ professional development behaviors. To inform the development of more effective fit strategies, the following future research directions are proposed: (a) examining perceived person–environment fit fluctuation patterns and relative factors, while considering the multidimensionality, measurement methods, and temporal variability of person–environment fit; (b) conducting research on person–vocation fit; (c) performing intervention studies, systematic reviews, or meta‐analysis to raise the level of evidence regarding the relationships between perceived person–environment fit and its antecedents and outcomes; and (d) identifying distinct fit profiles through cluster analysis and examining potential curvilinear relationships between fit dimensions and staff outcomes to deeply understand how nursing retention and well‐being are generated.

## Funding

This work was supported by the University of Tokyo under the “2024 Research Skill Enhancement Support for Female Researchers” program and by the JSPS KAKENHI Grant‐in‐Aid for Early‐Career Scientists (Grant Number: 25K20661).

## Conflicts of Interest

The authors declare no conflicts of interest.

## Supporting Information

Additional supporting information can be found online in the Supporting Information section.

## Supporting information


**Supporting Information** Supporting Information 1: Final search strategy. Supporting Information 2: Countries where the studies were conducted in (*n* = 45). Supporting Information 3: Studies included in the synthesis (*n* = 45).

## Data Availability

The data that support the findings of this study are available in the supporting information of this article.
