# Supplementary material for: Antecedents and Staff Outcomes Related to Multiple Dimensions of Perceived Person–Environment Fit Among Nurses: A Scoping Review
Source: J Nurs Manag. 2026 Aug 11;2026:2914169. doi: 10.1155/jonm/2914169 (PMC13462668; doi:10.1155/jonm/2914169)
Supplement: Supplementary file 1 [file JONM-2026-2914169-s001.docx]

| Supplementary Material 1. Final search strategy | | | |
| --- | --- | --- | --- |
| Database | Search strategy | | Number of studies |
| PubMed | **(((#1 OR #2 OR #3) AND #4) OR #5) AND #6** | | **274** |
|  | #1 | Search: “person”[All Fields] AND (“environment”[All Fields] OR “job”[All Fields] OR “organization”[All Fields] OR “supervisor”[All Fields] OR “group”[All Fields] OR “vocation”[All Fields] OR “occupation”[All Fields]) | 64,244 |
|  | #2 | Search: (“need”[All Fields] OR “needs”[All Fields]) AND (“supply”[All Fields] OR “supplies”[All Fields]) | 48,441 |
|  | #3 | Search: (“demand”[All Fields] OR “demands”[All Fields]) AND (“ability”[All Fields] OR “abilities”[All Fields]) | 19,128 |
|  | #4 | Search: (“fit”[All Fields] OR “fits”[All Fields] OR “congruenc*”[All Fields]) | 210,701 |
|  | #5 | Search: (“value”[All Fields] OR “values”[All Fields]) AND “congruenc*”[All Fields] | 15,830 |
|  | #6 | Search: (((“nurse's”[All Fields] OR “nurses”[MeSH Terms] OR “nurses”[All Fields] OR “nurse”[All Fields] OR “nurses's”[All Fields]) NOT “health nurse”[All Fields]) NOT “nurses, public health”[MeSH Terms]) | 465,664 |
| Note: Literature search performed on August 14, 2024. The other databases (Web of Science, CINAHL, and Ichushi-web) were searched using the same logical formula as for PubMed. | | | |

| Supplementary Material 2. Countries the studies were conducted in (n = 45) | |
| --- | --- |
| Countries | Number of studies |
| Canada | 8 |
| China | 7 |
| United States | 6 |
| Japan | 4 |
| South Korea | 3 |
| Australia | 2 |
| Indonesia | 2 |
| Nigeria | 2 |
| Taiwan | 2 |
| Thailand | 2 |
| Turkey | 2 |
| German | 1 |
| Ghana | 1 |
| Pakistan | 1 |
| Spain | 1 |
| Sweden | 1 |

| Supplementary Material 3. Studies included in the synthesis (n = 45) | | | | | | | | | | | | | | | | |
| --- | --- | --- | --- | --- | --- | --- | --- | --- | --- | --- | --- | --- | --- | --- | --- | --- |
| Authors, publication year | Country | Study design | Participants (valid response) | Examined PE fit | | Measurements | Analysis | Not causally related factors with PE fit | | | Work-related antecedents of PE fit | | Demographic-related antecedents of PE fit | | Outcomes of PE fit | |
|  |  |  |  | As stated in the literature | Classification in this review |  |  | Measured at the same time as PE fit | | Measured at different times as PE fit | Measured at the same time as PE fit | Measured before PE fit | Measured at the same time as PE fit | Measured before PE fit | Measured at the same time as PE fit | Measured after PE fit |
|  |  |  |  |  |  |  |  | Correlation | Categorical relationships | Correlation |  |  |  |  |  |  |
| (Afsar, 2016) [47] | Thailand | Longitudinal: Times 1 and 2 (12 months after T1) | 357 nurses and 77 doctors from three government hospitals ※doctors rated the "innovative work behavior" of nurses who were their subordinates | Person−organization fit | Person−organization fit | Resick et al. [31]: 5 items, x point scale (n/a)   * Measured at Times 1 and 2 | Correlations, structural equation model | (+) Knowledge sharing behavior (+) Doctor-rated innovative work behavior (+) Self-rated innovative work behavior | n/a | Measured before and after PO fit (×) Knowledge sharing behavior (×) Doctor-rated innovative work behavior (×) Self-rated innovative work behavior (×) Person−organization fit | n/a | n/a | n/a | n/a | (+) Knowledge sharing behavior (+) Doctor-rated innovative work behavior  (+) Self-rated innovative work behavior | n/a |
| (Afsar et al., 2018) [42] | Thailand | Cross-sectional | 441 nurses and 73 doctors from multiple hospitals ※doctors rated the "innovative work behavior" of nurses who were their subordinates | Person−organization fit | Person−organization fit | Cable and Judge [21]: 3 items, 5-point Likert-scale | Correlations, structural equation model | (+) Psychological empowerment (+) Sharing best practices (+) Sharing mistakes (+) Innovative work behavior (average of self-rated and doctor-rated) | n/a | n/a | n/a | n/a | n/a | n/a | (+) Psychological empowerment (+) Innovative work behavior (average of self-rated and doctor-rated) | n/a |
| (Akanni & Oduaran, 2022) [48] | Nigeria | Cross-sectional | 216 nurses from two federal teaching hospitals | Person−job fit | Person−job fit | Person−Job fit Scale from the Multidimensional Instrument of Person−Environment Fit [11]: 4 items, 7-point Likert scale | Correlation, mediation analyses | (+) Age (+) Cultural competence (+) Work−life balance (×) Job tenure | n/a | n/a | n/a | n/a | n/a | n/a | (+) Cultural competence (+) Work−life balance | n/a |
| (Anaza, 2015) [49] | United States | Cross-sectional | 139 practicing nurses | Person−job fit | Person−job fit | Saks and Ashforth [32]: 3 items, 5-point Likert-scale | Correlation, structural equation model | (+) Organizational identification  (+) Employee−customer identification (+) Person−organization fit | n/a | n/a | n/a | n/a | n/a |  | (+) Organizational identification (×) Employee−customer identification | n/a |
|  |  |  |  | Person−organization fit | Person−organization fit | Cable & Judge [21]: 3 items, 7 Likert-scale |  | (+) Organizational identification  (+) Employee-customer identification (+) Person-job fit | n/a | n/a | n/a | n/a | n/a |  | (+) Organizational identification (×) Employee−customer identification | n/a |
| (Atitsogbui & Amponsah-Tawiah, 2019) [50] | Ghana | Cross-sectional | 322 registered nurses from eight hospitals | Person−job fit | Person−job fit | Perceived Fit Scale [10]: 6 items, 5-point Likert-scale | Simple linear regression analysis, hierarchical multiple regression analysis | n/a | n/a | n/a | n/a | n/a | n/a |  | (×) Turnover intention (+) Psychological climate | n/a |
| (Boamah & Laschinger, 2016) [51] | Canada | Cross-sectional | 215 newly graduated nurses | Person−job match in the six areas of work−life | Person−job fit/match (in the areas of work−life) | The Areas of Worklife Scale (AWS) [24]:　18 items, 5-point Likert-scale  Score >3.0: A person−job match  Score <3.0: misfit between the worker and the workplace | Correlation, structural equation model | (-) Emotional exhaustion (-) Cynicism (-) Work−life interference (-) Job turnover | n/a | n/a | n/a | n/a | n/a |  | (-) Burnout | n/a |
| (Cao et al., 2021) [52] | China | Cross-sectional | 12,291 nurses in 23 hospitals | Person−job fit | Person−job fit | Person−Job Fit Scale from the Multidimensional Instrument of Person−Environment Fit [11]: 4 items, 7-point Likert-scale | T tests,  multilevel multiple logistic regression | n/a | Turnover intention (the score of person−job fit of the low level turnover intention group is higher than that of the high level turnover intention group) | n/a | n/a | n/a | n/a |  | (×) Turnover intention | n/a |
|  |  |  |  | Person−organization fit | Person−organization fit | Person−Organization Fit Scale from the Multidimensional Instrument of Person−Environment Fit [11]: 7 items, 7-point Likert-scale |  | n/a | Turnover intention (the score of person−organization fit of the low level turnover intention group is higher than that of the high level turnover intention group) | n/a | n/a | n/a | n/a |  | (-) Turnover intention | n/a |
|  |  |  |  | Person−supervisor fit | Person−supervisor fit | Person−Supervisor Fit Scale from the Multidimensional Instrument of Person−Environment Fit 5[11]: 5 items, 7-point Likert-scale |  | n/a | Turnover intention (the score of person−supervisor fit of the low level turnover intention group is higher than that of the high level turnover intention group) | n/a | n/a | n/a | n/a |  | (×) Turnover intention | n/a |
|  |  |  |  | Person−group fit | Person−group fit | Person−Group Fit Scale from the Multidimensional Instrument of Person−Environment Fit [11]: 10 items, 7-point Likert-scale |  | n/a | Turnover intention (the score of person−group fit of the low level turnover intention group is higher than that of the high level turnover intention group) | n/a | n/a | n/a | n/a |  | (-) Turnover intention | n/a |
| (Chu, 2021) [53] | Taiwan | Longitudinal: Times 1 and 2 (two months after Time 1) | 263 nurses working in major medical centers, regional hospitals, district hospitals, and clinics | Person−job fit | Person−job fit | Person−Job Fit Scale containing Demands−Abilities Fit and Needs−Supplies Fit [10]: 6 items, 6-point Likert-scale  * Measured at Time 1 | Correlation,  hierarchical regression analysis | (-) Compassion fatigue | n/a | Variables measured after PJ fit (+) Job performance (+) Organizational citizenship behavior | n/a | n/a | n/a |  | n/a | (+) Job performance (+) Organizational citizenship behavior |
| (Dotson et al., 2012) [54] | United States | Cross-sectional | 1,046 nurses | Value congruence (i.e., the level of alignment between the values of the nurses and the values they perceive exist in their organization) | Value congruence | Developed a scale; 3 items, x-point scale (n/a) | Structural equation model | n/a | n/a | n/a | n/a | n/a | n/a |  | (+) Behavioral intentions to take a rural job | n/a |
| (Dotson et al., 2014) [55] | United States | Cross-sectional | 861 registered nurses | Value congruence (i.e., the nurse’s values fit with those of the employer) | Value congruence | Developed a scale: 3 items, x-point scale (n/a) | Structural equation model | n/a | n/a | n/a | n/a | n/a | n/a |  | (-) Behavioral intentions to leave a current job (+) Behavioral intentions to leave the nursing profession (+) Job satisfaction | n/a |
| (Enwereuzor et al., 2018) [56] | Nigeria | Cross-sectional | 224 staff nurses from four hospitals | Person−job fit | Person−job fit | Person−Job Fit Scale from the Multidimensional Instrument of Person−Environment Fit [11]: x items, 5-point Likert-scale | Correlation, moderated multiple regression | (+) Gender ※0 = male, 1 = female (+) Transformational leadership (+) Work engagement (×) Marital status ※0 = single, 1 = married (×) Organizational tenure (×) Age | n/a | n/a | n/a | n/a | n/a |  | (+) Work engagement | n/a |
| (Gascon et al., 2013) [57] | Spain | Cross-sectional | 878 nursing professions (only permanent staff working continuously in the same post for at least one year) in three hospitals and 22 rural and urban primary care centers | (Mis)matches in values (areas of work−life) | Value congruence | Values Scale of The Areas of Worklife Scale, measuring the perceived congruence employed in different areas of their work [24]: x items, 5-point Likert-scale | Regression analysis | n/a | n/a | n/a | n/a | n/a | n/a |  | (+) Efficacy  (+) Energy (+) Involvement | n/a |
| (Greco et al., 2006) [58] | Canada | Cross-sectional | 322 registered nurses (RNs) working full-time or part-time in acute care hospitals in Ontario | Overall person−job fit (overall areas of work−life) | Person−job fit/match (in the areas of work−life) | The Areas of Worklife Scale [27]: 29 items, 5-point Likert-scale | Structural equation model | n/a | n/a | n/a | (+) Structural empowerment | n/a | n/a |  | (-) Emotional exhaustion | n/a |
| (Hylén et al., 2018) [59] | Sweden | Cross-sectional | 353 nursing staff (i.e., registered nurses and assistant nurses) in 10 inpatient psychiatric wards in central Sweden | Value incongruence (i.e., the difference between personal value hierarchy, and the perceived value hierarchy of a reference group or organization) | Value congruence | Developed an item: one item, a visual analogue scale (132 mm, ranging from “I do not agree” to “I fully agree”; P1 = 0−44 mm, P2 = 45−90 mm, P3 = 91−132 mm)  1. I feel that those in my workplace/in our organization have different values than I have | Kruskal–Wallis analysis, Dunn’s post hoc analysis | n/a | Social interaction (the median of the value congruence group is higher than that of the value middle congruence group and the incongruence group) Leadership (the median of the value congruence group is higher than that of the value middle congruence group and the incongruence group) Organizational culture and climate (the median of the value congruence group is higher than that of the value middle congruence group and the incongruence group) | n/a | n/a | n/a | n/a |  | n/a | n/a |
| (Inoue, Kunie, Takemura, Ichikawa, et al., 2021) [20] | Japan | Cross-sectional | 209 nurses (registered nurses, assistant nurses, midwives) | Needs−supplies fit | Needs−supplies fit | Needs−Supplies Fit Scale of the Japanese version of the Perceived Fit Scale [10,20], 3 items, 7-point Likert | Correlation | (+) Affective organizational commitment (+) Job satisfaction (+) Organization identification (+) Self efficacy (+) Sense of unity (+) Sense of meaning  (+) Sense of patient impact (+) Sense of grasp (+) Structural empowerment | n/a | n/a | n/a | n/a | n/a |  | n/a | n/a |
|  |  |  |  | Demands−abilities fit | Demands−abilities fit | Demands-Abilities Fit Scale of the Japanese version of the Perceived Fit Scale [10,20], 3 items, 7-point Likert scale | Correlation | (+) Affective organizational commitment (+) Job satisfaction (+) Organization identification (+) Self efficacy (+) Sense of unity (+) Sense of meaning  (+) Sense of patient impact (+) Sense of grasp (+) Structural empowerment | n/a | n/a | n/a | n/a | n/a |  | n/a | n/a |
|  |  |  |  | Person−organization fit | Person−organization fit | Person−Organization Fit Scale of the Japanese version of the Perceived Fit Scale [10,20], 3 items, 7-point Likert scale | Correlation | (+) Affective organizational commitment (+) Job satisfaction (+) Organization identification (+) Self efficacy (+) Sense of unity (+) Sense of meaning  (+) Sense of patient impact (+) Sense of grasp (+) Structural empowerment | n/a | n/a | n/a | n/a | n/a |  | n/a | n/a |
|  |  |  |  | Person−supervisor fit | Person−supervisor fit | Person−Supervisor Fit Scale of the Japanese version of the Perceived Fit Scale [10,20], 3 items, 7-point Likert scale | Correlation | (+) Affective organizational commitment (+) Job satisfaction (+) Organization identification (+) Sense of unity (+) Sense of meaning  (+) Sense of patient impact (+) Sense of grasp (+) Structural empowerment (×) Self efficacy | n/a | n/a | n/a | n/a | n/a |  | n/a | n/a |
|  |  |  |  | Person−group fit | Person−group fit | Person−Group Fit Scale of the Japanese version of the Perceived Fit Scale [10,20], 3 items, 7-point Likert scale | Correlation | (+) Affective organizational commitment (+) Job satisfaction (+) Organization identification (+) Sense of unity (+) Sense of meaning  (+) Sense of patient impact (+) Sense of grasp (+) Structural empowerment (×) Self efficacy | n/a | n/a | n/a | n/a | n/a |  | n/a | n/a |
| (Inoue, Kunie, Takemura, Kida, et al., 2021) [14] | Japan | Longitudinal: Times 1 and 2 (eight months after Time 1) | 324 nurses (registered nurses, assistant nurses, and midwives) of two hospitals | Needs−supplies fit | Needs−supplies fit | Needs−Supplies Fit Scale of the Japanese version of the Perceived Fit Scale [10,20], 3 items, 7-point Likert scale  *Measured at Time 1 and 2; Dependent variable is a value at Time 2. | Correlation, simple linear regression analysis, multiple linear regression analysis | (-) Operational changes | n/a | Variables measured before NS fit (+) Communication about nursing practice with colleagues (+) Learning in hospitals (+) Learning outside hospitals (+) Needs−supplies fit (+) On-the-job opportunities for professional growth | (×) Operational changes | Simple linear regression analysis (+) Learning in hospitals (+) Learning outside hospitals (+) Communication about nursing practice with colleagues (+) On-the-job opportunities for professional growth  Multiple linear regression analysis (+) Needs−supplies fit (+) On-the-job opportunities for professional growth (×) Communication about nursing practice with colleagues (×) Learning in hospitals (×) Learning outside hospitals |  | Multiple linear regression analysis (×) Employment position (×) Nursing unit (×) Sex (×) Total years in current nursing unit (×) Total years of nursing experience | n/a | n/a |
|  |  |  |  | Demands−abilities fit | Demands−abilities fit | Demands−Abilities Fit Scale of the Japanese version of the Perceived Fit Scale [10,20], 3 items, 7-point Likert scale  *Measured at Times 1 and 2; dependent variable is a value at Time 2 |  | (-) Operational changes | n/a | Variables measured before DA fit (+) Communication about nursing practice with colleagues (+) Demands− abilities fit (+) Learning in hospitals (+) Learning outside hospitals (+) On-the-job opportunities for professional growth | (-) Operational changes | Simple linear regression analysis (+) Learning in hospitals (+) Learning outside hospitals (+) Communication about nursing practice with colleagues (+) On-the-job opportunities for professional growth  Multiple linear regression analysis (+) Demands−abilities fit (+) On-the-job opportunities for professional growth  (×) Communication about nursing practice with colleagues (×) Learning in hospitals (×) Learning outside hospitals |  | Multiple linear regression analysis (×) Employment position (×) Nursing unit (×) Sex (×) Total years in the current nursing unit (×) Total years of nursing experience | n/a | n/a |
|  |  |  |  | Person−organization fit | Person−organization fit | Person−Organization Fit Scale of the Japanese version of the Perceived Fit Scale [10,20], 3 items, 7-point Likert scale  *Measured at Times 1 and 2; dependent variable is a value at Time 2 |  | (-) Operational changes | n/a | Variables measured before PO fit (+) Communication about nursing practice with colleagues (+) Learning in hospitals (+) Learning outside hospitals (+) On-the-job opportunities for professional growth (+) Person−organization fit | (×) Operational changes | Simple linear regression analysis (+) Communication about nursing practice with colleagues (+) Learning in hospitals (+) Learning outside hospitals (+) On-the-job opportunities for professional growth  Multiple linear regression analysis (+) On-the-job opportunities for professional growth (+) Person−organization fit  (×) Communication about nursing practice with colleagues (×) Learning in hospitals (×) Learning outside hospitals |  | Multiple linear regression analysis (×) Employment position (×) Nursing unit (×) Sex (×) Total years in the current nursing unit (×) Total years of nursing experience | n/a | n/a |
|  |  |  |  | Person−group fit | Person−group fit | Person−Group Fit Scale of the Japanese version of the Perceived Fit Scale [10,20], 3 items, 7-point Likert scale  *Measured at Times 1 and 2; dependent variable is a value at Time 2 |  | n/a | n/a | Variables measured before PG fit (+) Communication about nursing practice with colleagues (+) Learning in hospitals (+) Learning outside hospitals (+) On-the-job opportunities for professional growth (+) Person−group fit | (×) Operational changes | Simple linear regression analysis (+) Communication about nursing practice with colleagues (+) Learning in hospitals (+) Learning outside hospitals (+) On-the-job opportunities for professional growth  Multiple linear regression analysis (+) On-the-job opportunities for professional growth (+) Person−group fit  (×) Communication about nursing practice with colleagues (×) Learning in hospitals (×) Learning outside hospitals |  | Multiple linear regression analysis (×) Employment position (×) Nursing unit (×) Sex (×) Total years in current nursing unit (×) Total years of nursing experience | n/a | n/a |
| (Inoue et al., 2023) [60] | Japan | Cross-sectional | 662 nurses (i.e., registered nurses, assistant nurses, and midwives) from three hospitals | Needs−supplies fit | Needs−supplies fit | Needs−Supplies Fit Scale of the Japanese version of the Perceived Fit Scale [10,20], 3 items, 7-point Likert scale | Correlation, t-test, one-way analysis of variance,  multiple linear regression analysis | (+) Demands−abilities fit (+) Perceived reflection support from colleagues (+) Perceived reflection support from nurse managers (+) Person−organization fit (+) Person−supervisor fit (+) Person−group fit  (×) Total years of nursing experience (×) Total years in the current nursing unit | Employment position (the score of needs−supplies fit of senior staff is higher than that of staff) Operational changes (the score of needs−supplies fit of the many operational changes group is lower than that of others)  (×) Hospital (×) Nursing unit (×) Sex (×) Qualification | n/a | (+) Perceived reflection support from colleagues (+) Perceived reflection support from nurse managers  (×) Operational changes | n/a | (×) Employment position (×) Hospital (×) Nursing unit (×) Sex (×) Total years in the current nursing unit (×) Total years of nursing experience |  | n/a | n/a |
|  |  |  |  | Demands−abilities fit | Demands−abilities fit | Demands−Abilities Fit Scale of the Japanese version of the Perceived Fit Scale [10,20], 3 items, 7-point Likert scale |  | (+) Needs−supplies fit (+) Perceived reflection support from colleagues (+) Perceived reflection support from nurse managers (+) Person−organization fit (+) Person−supervisor fit (+) Person−group fit (+) Total years in the current nursing unit  (×) Total years of nursing experience | Employment position (the score of demands−abilities fit of senior staff is higher than that of staff) Operational changes (the score of demands−abilities fit of the many operational changes group is lower than that of others)  (×) Hospital (×) Nursing unit (×) Sex (×) Qualification | n/a | (+) Perceived reflection support from colleagues (+) Perceived reflection support from nurse managers  (-) Operational changes | n/a | (×) Employment position (×) Hospital (×) Nursing unit (×) Sex (×) Total years in the current nursing unit (×) Total years of nursing experience |  | n/a | n/a |
|  |  |  |  | Person−organization fit | Person−organization fit | Person−Organization Fit Scale of the Japanese version of the Perceived Fit Scale [10,20], 3 items, 7-point Likert scale |  | (+) Demands−abilities fit (+) Needs−supplies fit (+) Perceived reflection support from colleagues (+) Perceived reflection support from nurse managers (+) Person−supervisor fit (+) Person−group fit   (×) Total years in the current nursing unit (×) Total years of nursing experience | Nursing unit (the score of person−organization fit of the inpatient unit is higher than that of the operating room) Hospital (the score of person−organization fit of Hospital 3 is higher than that of Hospitals 1 and 2) Operational changes (the score of person−organization fit of the many operational changes group is lower than that of others)  (×) Employment position (×) Sex (×) Qualification | n/a | (+) Perceived reflection support from colleagues (+) Perceived reflection support from nurse managers  (×) Operational changes | n/a | (+) Total years of nursing experience (+) Hospital (Hospital 2 < Hospital 3)  (×) Employment position (×) Nursing unit (×) Sex (×) Total years in the current nursing unit |  | n/a | n/a |
|  |  |  |  | Person−supervisor fit | Person−supervisor fit | Person−Supervisor Fit Scale of the Japanese version of the Perceived Fit Scale [10,20], 3 items, 7-point Likert scale |  | (+) Demands−abilities fit (+) Needs−supplies fit (+) Perceived reflection support from colleagues (+) Perceived reflection support from nurse managers (+) Person−organization fit (+) Person−group fit  (-) Total years of nursing experience  (×) Total years in the current nursing unit | Nursing unit (the score of person−supervisor fit of the inpatient unit is higher than that of the outpatient unit and others) Operational changes (the score of person−supervisor fit of the many operational changes group is lower than that of others)  (×) Employment position (×) Hospital (×) Sex (×) Qualification | n/a | (+) Perceived reflection support from nurse managers  (-) Operational changes  (×) Perceived reflection support from colleagues | n/a | (×) Employment position (×) Hospital (×) Nursing unit (×) Sex (×) Total years in the current nursing unit (×) Total years of nursing experience |  | n/a | n/a |
|  |  |  |  | Person−group fit | Person−group fit | Person−Group Fit Scale of the Japanese version of the Perceived Fit Scale [10,20], 3 items, 7-point Likert scale |  | (+) Demands−abilities fit (+) Needs−supplies fit (+) Perceived reflection support from colleagues (+) Perceived reflection support from nurse managers (+) Person−organization fit (+) Person−supervisor fit  (-) Total years of nursing experience  (×) Total years in the current nursing unit | Nursing unit (the score of person−group fit of the inpatient and outpatient units and others is higher than that of the operating room) Hospital (the score of person−group fit of Hospital 3 is higher than that of Hospital 1) Operational changes (the score of person−group fit of the many operational changes group is lower than that of others)  (×) Employment position (×) Sex (×) Qualification | n/a | (+) Perceived reflection support from colleagues (+) Perceived reflection support from nurse managers  (×) Operational changes | n/a | (-) Nursing unit (inpatient > operating room)  (×) Employment position (×) Hospital (×) Sex (×) Total years in the current nursing unit (×) Total years of nursing experience |  | n/a | n/a |
| (Iqbal et al., 2020) [43] | Indonesia | Cross-sectional | 138 nurses from two government hospitals | Person−organization fit | Person−organization fit | Cable and Judge [21]: 3 items, 5-point Likert-scale | Correlations, structural equation model, multiple regression and factor analysis | (+) Workplace spirituality (+) Work engagement | n/a | n/a | s | n/a | n/a |  | (+) Work engagement | n/a |
| (Kakar et al., 2022) [61] | Pakistan | Cross-sectional | 331 nurses from three public sector hospitals | Person−job fit | Person−job fit | The Overall Person−Job Fit Scale of the Perceived Fit Scale [10]: 6 items, 5-point Likert scale | Correlations, structural equation model | (+) Work−life balance practices (-) Organizational cynicism | n/a | n/a | (+) Work−life balance practices | n/a | n/a |  | (-) Organizational cynicism | n/a |
| (Spence Laschinger et al., 2006) [19] | Canada | Cross-sectional | 322 registered nurses working full-time or part-time in acute-care hospitals | Job–person fit or match (areas of work−life) | Person−job fit/match (in the areas of work−life) | The Areas of Worklife Scale measuring six areas of work−life (i.e., workload, control, rewards, community, fairness, values) [27]: 29 items, 5-point Likert scale  Score >3.0: A person−job match  Score <3.0: A person−job mismatch | Structural equation modeling | n/a | n/a | n/a | (+) Empowerment (workload, control, rewards, community, fairness, and values that are the areas of work−life) | n/a | n/a |  | (+) (Workload→)emotional exhaustion (-) (Fairness, community, value→)emotional exhaustion (+) (Control→)workload, fairness, reward, community | n/a |
| (Laschinger & Grau, 2012) [62] | Canada | Cross-sectional | 165 nurses with experience in nursing of one year or less | Person–job fit (areas of work–life) | Person–job fit/match (in the areas of work–life) | The Areas of Worklife Scale [24]: 20 items, 5-point Likert scale | Correlations, structural equation model | (+) Psychological capital  (-) Bullying experiences (-) Cynicism (-) Emotional exhaustion (-) Physical health (-) Mental health | n/a | n/a | (+) Psychological capital | n/a | n/a |  | (-) Bullying experiences (-) Emotional exhaustion | n/a |
| (Laschinger et al., 2015) [63] | Canada | Cross-sectional | 1,009 newly graduated nurses with less than three years of nursing experience working in direct patient care settings | Areas of work–life match (person–job fit) | Person–job fit/match (in the areas of work–life) | The Areas of Worklife Scale [26]: 18 items, 5-point Likert scale | Correlations, structural equation model | (+) Authentic leadership (+) Mental health (positive) (+) Occupational coping self-efficacy  (-) Cynicism (-) Emotional exhaustion (-) Interpersonal strain | n/a | n/a | (+) Authentic leadership | n/a | n/a |  | (+) Occupational coping self-efficacy | n/a |
| (Laschinger & Read, 2016) [64] | Canada | Cross-sectional | 993 newly graduated nurses randomly selected from the nursing registry bodies of 10 Canadian provinces | Person–job fit with six areas of work–life | Person–job fit/match (in the areas of work–life) | The Areas of Worklife Scale [23]: 20 items, 5-point Likert scale | Correlations, structural equation model | (+) Authentic leadership (+) Civility norms  (-) Burnout (-) Coworker incivility | n/a | n/a | (+) Authentic leadership | n/a | n/a |  | (+) Civility norms | n/a |
| (Lee et al., 2024) [40] | South Korea | Longitudinal: Times 1 and 2 (15 months after Time 1) | 586 newly graduated nurses; T1 (2020.11-12) senior nursing students T2 (2022.2-3) working as newly graduated nurses in hospitals | Person–job fit | Person–job fit | Person−Job Fit Perception Scale [33]: 8 items (items of the Person−Job Fit Perception Scale and Person−Organization Fit Perception Scale), 5-point Likert scale  *Measured at Time 2 | Differences between the retention and turnover groups were assessed by t-tests and χ2 tests | n/a | Retention/Turnover (the score of person−job fit of the retention group is higher than that of the turnover group) | n/a | n/a | n/a | n/a |  | (+) Retention | n/a |
|  |  |  |  | Person–organization fit | Person–organization fit | Person−Organization Fit Perception Scale [33]: 8 items (items of the Person−Job Fit Perception Scale and Person−Organization Fit Perception Scale), 5-point Likert scale  *Measured at Time 2 |  | n/a | Retention/Turnover (the score of person−organization fit of the retention group is higher than that of the turnover group) | n/a | n/a | n/a | n/a |  | n/a | n/a |
| (Leiter, 2008) [65] | Canada | Cross-sectional | 725 nurses from acute care facilities | Value congruence | Value congruence | Value Scale of The Areas of Worklife Scale [24]: 5 items, 5-point Likert scale  Score >3.0: Congruent  Score <3.0: Incongruent | Correlations, Structural equation model | (+) Control (+) Efficacy (+) Fairness (+) Workload (+) Organizational rating (participants evaluated three issues regarding the extent to which they had changed in the past year; the specific issues were workload, hours of work, and workplace health)  (-) Cynicism (-) Exhaustion | n/a | n/a | (+) Fairness | n/a | n/a |  | (+) Efficacy (+) Organizational rating   (-) Cynicism (-) Exhaustion | n/a |
| (Leiter et al., 2009) [66] | Canada | Cross-sectional | 255 Generation X nurses and 193 Baby Boomer nurses from acute care facilities | Value congruence | Value congruence | Value Scale of The Areas of Worklife Scale [25]: x item, 5-point Likert scale | Correlations, MANOVA, multiple regressions | (+) Control (+) Efficacy (+) Personal knowledge sharing  (-) Cynicism (-) Exhaustion (-) Turnover intent | n/a | n/a | n/a | n/a | (+) Generation  (×) Tenure |  | n/a | n/a |
| (Li et al., 2020) [41] | China | Cross-sectional | 1,313 full-time registered nurses who have less than three years of work experience, from 23 hospitals | Person–job fit | Person–job fit | Person−Job Fit Scale from the Perceived Person−Environment Fit Scale [11]: 4 items, 7-point Likert scale | Wilcoxon test, multilevel logistic regression analysis | (-) Intention to leave | n/a | n/a | n/a | n/a | n/a |  | (+) Possibility of access to external work  (-) Motivation in finding other employment (-) Possibility of quitting the present job  (×) Turnover intention | n/a |
|  |  |  |  | Person–organization fit | Person–organization fit | Person−Organization Fit Scale from the Perceived Person−Environment Fit Scale [11]: 7 items, 7-point Likert scale |  | (-) Intention to leave | n/a | n/a | n/a | n/a | n/a |  | (-) Motivation in finding other employment (-) Possibility of access to external work (-) Possibility of quitting the present job (-) Turnover intention | n/a |
|  |  |  |  | Person–supervisor fit | Person–supervisor fit | Person−Supervisor Fit Scale from the Perceived Person−Environment Fit Scale [11]: 5 items, 7-point Likert scale |  | (-) Intention to leave | n/a | n/a | n/a | n/a | n/a |  | (-) Possibility of quitting the present job  (×) Motivation in finding other employment (×) Possibility of access to external work (×) Turnover intention | n/a |
|  |  |  |  | Person–group fit | Person–group fit | Person−Group Fit Scale from the Perceived Person−Environment Fit Scale [11]: 10 items, 7-point Likert scale |  | (-) Intention to leave | n/a | n/a | n/a | n/a | n/a |  | (×) Motivation in finding other employment (×) Possibility of quitting the present job (×) Possibility of access to external work (×) Turnover intention | n/a |
| (Li et al., 2023) [67] | China | Longitudinal: Times 1 and 2 (three months after Time 1) | 695 nurses from three hospitals | Person–job fit | Person–job fit | Lauver and Kristof-Brown [22]: 4 items, 7-point Likert scale  *Measured at Time 1 | Correlations, hierarchical regression analysis | (+) Digital competence (+) Self efficacy  (-) Working years   (×) Age (×) Gender | n/a | Variables measured after PJ fit (+) Workplace wellbeing | (+) Digital competence (+) Self efficacy | n/a | (×) Age (×) Gender (×) Working years |  | n/a | (+) Workplace wellbeing |
| (Lim et al., 2018) [68] | South Korea | Cross-sectional | 348 nurses from five general hospitals | Value congruence | Value congruence | Korean version of the Value Congruence Scale [29,30]: x items, 5-point Likert scale  ※Value congruence refers to the homogeneity of values and identities that are held by the team leader and team members as an effect of the members’ perceived self-concepts in their relationships with the leader [29] | Correlations, structural equation model | (+) Leader emotional intelligence (+) Leader trust (+) Organizational citizenship behavior (+) Self-emotional intelligence | n/a | n/a | (+) Leader's emotional intelligence (+) Leader trust  (-) Self-emotional intelligence | n/a | n/a |  | (×) Organizational citizenship behavior | n/a |
| (Liu et al., 2023) [69] | China | Cross-sectional | 326 registered nurses from hospitals | Person–organization fit | Person–organization fit | Person−Organization Fit Scale [35]: 40 items, 5-point Likert scale  Subscales value fit (22 items), personal demand–organizational supply (10 items), and personal ability–organizational demand (8 items) | T-test,  one-way ANOVA, correlation,  moderated multiple regression | (+) Emotional intelligence (+) Transformational leadership | Education (the score of person−organization fit of an individual with a master's degree is lower than that of those with other degrees) Sex (the score of person−organization fit of women is higher than that of men) | n/a | (+) Emotional intelligence (+) Transformational leadership | n/a | (-/×) Education (-/×) Gender |  | n/a | n/a |
| (Martadiani et al., 2022) [44] | Indonesia | Cross-sectional | 311 nurses at a private hospital | Person–job fit | Person–job fit | Cable and DeRue [10]: 6 items, x-point Likert scale | Logistic regression analysis | n/a | n/a | n/a | n/a | n/a | n/a |  | (-) Turnover intention (+) Job satisfaction | n/a |
|  |  |  |  | Person–organization fit | Person–organization fit | Cable and DeRue [10] and Supelli and Creed [34]: 10 items, x-point Likert scale |  | n/a | n/a | n/a | n/a | n/a | n/a |  | (-) Turnover intention (+) Job satisfaction | n/a |
| (Nagaoka et al., 2023) [38] | Japan | Cross-sectional | 148 registered nurses, public health nurses, and midwives, excluding nurse managers, from eight hospitals, who had been at their new workplace for five months | Needs–supplies fit | Needs–supplies fit | Needs−Supplies Fit Scale of the Japanese version of the Perceived Fit Scale [10,20], 3 items, 7-point Likert scale | Simple regression analysis, multiple regression analysis | n/a | (×) Prior workplace (no significant score difference was found between nurses who transferred to another department within the same hospital and those recently hired by their current hospital) | n/a | 1. Simple regression analysis (+) Assignment to the nurses’ department of choice; as requested (Ref = Others)  Nurse managers’ support: (+) Emotional support (+) Appraisal support (+) Information support (+) Instrumental support  Colleagues' support (+) Emotional support (+) Appraisal support (+) Information support (+) Instrumental support  On-the-job training:  (+) Teaching (+) Consultation  Individual　learning behaviors (+) Learning from practice (+) Learning from feedback (×) Learning through reflection  (×) Learning from others  (×) Learning from training   2. Multiple regression (+) Assignment to the nurses’ department of choice; as requested (+) Nurse managers' informational support | n/a | Multiple regression (×) Employment position (×) Sex (×) Number of prior affiliated facilities (×) Total years of nursing experience (×) Qualification (×) Recently hired with prior clinical experience |  | n/a | n/a |
|  |  |  |  | Demands–abilities fit | Demands–abilities fit | Demands−Abilities Fit scale of the Japanese version of the Perceived Fit Scale [10,20], 3 items, 7-point Likert scale |  | n/a | (×) Prior workplace (no significant score difference was found between nurses who transferred to another department within the same hospital and those recently hired by their current hospital) | n/a | 1. Simple regression analysis (+) Assignment to the nurses’ department of choice; as requested (Ref = Others) Nurse managers’ support: (+) Emotional support (+) Appraisal support (+) Information support (+) Instrumental support  Colleagues' support (+) Emotional support (+) Appraisal support (+) Information support (+) Instrumental support  On-the-job training:  (+) Teaching (+) Consultation  Individual　learning behaviors (+) Learning through reflection (+) Learning from practice (+) Learning from feedback (+) Learning from others (×) Learning from training  2. Multiple regression analysis (+) Assignment to the nurses’ department of choice; as requested (+) Nurse managers' appraisal support (+) Colleagues' emotional support | n/a | Multiple regression (×) Employment position (×) Number of prior affiliated facilities (×) Qualification (×) Recently hired with prior clinical experience (×) Sex (×) Total years of nursing experience |  | n/a | n/a |
|  |  |  |  | Person–organization fit | Person–organization fit | Person−Organization Fit Scale of the Japanese version of the Perceived Fit Scale [10,20], 3 items, 7-point Likert scale |  | n/a | (×) Prior workplace (no significant score difference was found between nurses who transferred to another department within the same hospital and those recently hired by their current hospital) | n/a | 1. Simple regression analysis Nurse managers’ support: (+) Emotional support (+) Appraisal support (+) Information support (+) Instrumental support  Colleagues' support (+) Emotional support (+) Appraisal support (+) Information support (×) Instrumental support  On-the-job training:  (+) Teaching (+) Consultation  Individual　learning behaviors (+) Learning from feedback (+) Learning from others (×) Learning through reflection (×) Learning from practice (×) Learning from training  (×) Assignment to the nurses’ department of choice  2. Multiple regression analysis (+) Nurse managers' informational support | n/a | Multiple regression (×) Employment position (×) Number of prior affiliated facilities (×) Qualification (×) Recently hired with prior clinical experience (×) Sex (×) Total years of nursing experience |  | n/a | n/a |
|  |  |  |  | Person–supervisor fit | Person–supervisor fit | Person−Supervisor Fit Scale of the Japanese version of the Perceived Fit Scale [10,20], 3 items, 7-point Likert scale |  | n/a | (×) Prior workplace (no significant score difference was found between nurses who transferred to another department within the same hospital and those recently hired by their current hospital) | n/a | 1. Simple regression analysis Nurse managers’ support: (+) Emotional support (+) Appraisal support (+) Information support (+) Instrumental support  Colleagues' support (+) Emotional support (+) Appraisal support (+) Information support (+) Instrumental support  On-the-job training:  (+) Teaching (+) Consultation  Individual　learning behaviors (+) Learning through reflection (+) Learning from practice (+) Learning from feedback (+) Learning from others (×) Learning from training  (×) Assignment to the nurses’ department of choice  2. Multiple regression analysis (+) Nurse managers' emotional support (+) Nurse managers' appraisal support (+) Individual　learning behaviors: Learning from practice | n/a | Multiple regression (×) Employment position (×) Number of prior affiliated facilities (×) Qualification (×) Recently hired with prior clinical experience (×) Sex (×) Total years of nursing experience |  | n/a | n/a |
|  |  |  |  | Person–group fit | Person–group fit | Person−Group Fit Scale of the Japanese version of the Perceived Fit Scale [10,20], 3 items, 7-point Likert scale |  | n/a | (×) Prior workplace (no significant score difference was found between nurses who transferred to another department within the same hospital and those recently hired by their current hospital) | n/a | 1. Simple regression analysis Nurse managers’ support: (+) Emotional support (+) Appraisal support (+) Information support (+) Instrumental support  Colleagues' support (+) Emotional support (+) Appraisal support (+) Information support (+) Instrumental support  On-the-job training:  (+) Teaching (+) Consultation  Individual　learning behaviors (+) Learning from practice (+) Learning from feedback (+) Learning from others (×) Learning from reflection (×) Learning from training  (×) Assignment to the nurses’ department of choice  2. Multiple regression analysis (+) Nurse managers’ support: Appraisal support (+) On-the-job training: Consultation | n/a | Multiple regression (×) Employment position (×) Number of prior affiliated facilities (×) Qualification (×) Recently hired with prior clinical experience (×) Sex (×) Total years of nursing experience |  | n/a | n/a |
| (Newton et al., 2013) [18] | Australia | Cross-sectional | 252 nurses who were at least 18 years old in age and residing in Australia | Subjective fit (how well employees perceive their own characteristics to match the organization) | Person–organization fit | Cable and DeRue [10] : 3 items, 5-point Likert scale | Correlations, path model analysis | (+) Flexibility-limiting change (+) Flexibility-promoting change (+) Job satisfaction (+) Organizational commitment (+) Psychological health  (-) Administrative stressors | n/a | n/a | (+) Flexibility-promoting change  (-) Flexibility-limiting change | n/a | n/a |  | (+) Job satisfaction  (+) Organizational commitment  (-) Administrative stressors | n/a |
| (Özlü & Türe, 2024) [70] | Turkey | Cross-sectional | 500 nurses working in a university hospital | Person–organization fit | Person–organization fit | Person−Organization Fit Scale Netemeyer et al. [28]: 4 items, x-point Likert scale | Structural equation model | n/a | n/a | n/a | (+) Perceived organizational support | n/a | n/a |  | (×) Perceived job stress | n/a |
| (Park et al., 2022) [71] | South Korea | Cross-sectional | 467 new graduate nurses working in hospitals | Person–job fit | Person–job fit | Lauver and Kristof-Brown [22]: 2 items, 5-point Likert scale | Chi-square statistics, multivariate logistic regression | n/a | Difficulty in adapting (the score of PJ fit of the no difficulty in adapting group is higher than that of the difficulty in adapting group) Intent to leave job or organization (the score of PJ fit of intent to leave group is higher than that of the no intent to leave group) | n/a | n/a | n/a | n/a |  | (-) Difficulty in adapting  (×) Intent to leave job or organization | n/a |
| (Peng et al., 2014) [72] | Taiwan | Cross-sectional | 349 registered nurses working at two hospitals | Needs–supplies fit | Needs–supplies fit | Cable and DeRue [10] : 3 items, 6-point Likert scale | Correlation, hierarchical multiple regression | (+) Demand−abilities fit (+) Person−organization fit (+) Work engagement  (-) Turnover intention  (×) Education (×) Tenure | n/a | n/a | n/a | n/a | n/a |  | (+) Work engagement  (-) Turnover intention | n/a |
|  |  |  |  | Demands–abilities fit | Demands–abilities fit | Cable and DeRue [10] : 3 items, 6-point Likert scale |  | (+) Needs−supplies fit (+) Person−organization fit (+) Tenure (+) Work engagement  (-) Turnover intention  (×) Education | n/a | n/a | n/a | n/a | n/a |  | (×) Turnover intention | n/a |
|  |  |  |  | Person–organization fit | Person–organization fit | Cable and DeRue [10] : 3 items, 6-point Likert scale |  | (+) Demands−abilities fit (+) Needs−supplies fit (+) Work engagement  (-) Turnover intention  (×) Education (×) Tenure | n/a | n/a | n/a | n/a | n/a |  | (+) Work engagement  (-) Turnover intention | n/a |
| (Rahnfeld et al., 2023) [45] | Germany | Longitudinal: Times 1 and 2 (12 months after Time 1) | 222 nurses in 44 care services (18 home care and 26 nursing homes) | Person–organization fit | Person–organization fit | Kristof [13]: 4 items, 7-point Likert scale  *Measured at Time 1 | Correlation, hierarchical multiple regression | (+) Health (+) Job control (+) Supervisor support (+) Task identity  (-) Exhaustion (-) Depressive mood (-) Sex (-) Social conflict  (×) Age (×) Contract (×) Qualification (×) Leadership (×) Working hours (×) Time pressure | n/a | Variables measured after PO fit (+) Health  (-) Depressive mood (-) Exhaustion (-) Intention to leave the organization (-) Intention to leave the profession | n/a | n/a | n/a |  | n/a | (×) Exhaustion means between Times 1 and 2 (×) Intention to leave the organization (×) Intention to leave the profession |
| (Ren & Hamann, 2015) [73] | United States | Cross-sectional | 504 nursing employees (84 registered nurses, 115 licensed practical nurses, 305 certified nursing assistants) at 35 nursing homes  ※The results from the group that consisted of registered nurses and licensed practical nurses were only reported in this table and the results of this manuscript | Value congruence (between employees and the organization) | Value congruence | Developed items: 2 items, 5-point Likert scale | Structural equation model | n/a | n/a | n/a | n/a | n/a | n/a |  | (+) Job satisfaction (+) Organizational commitment  (×) Turnover intention | n/a |
| (Risman et al., 2016) [74] | United States | Cross-sectional | 753 full-time registered nurses employed in a Midwestern hospital system | Value congruence (person–organization) | Value congruence | Perceived Value Congruence Scale of the Areas of Worklife Scale [24]: x items, 4-point Likert scale | Correlation, ordinary least squares regression | (+) Job satisfaction (+) Patient care quality  (×) Hours per week (×) Patient load (×) Patient acuity | n/a | n/a | n/a | n/a | n/a |  | (+) Job satisfaction (+) Patient care quality | n/a |
| (Shao et al., 2018) [75] | China | Cross-sectional | 19,149 nurses working in all wards from Level 1(<300 beds), Level 2 (300–500 beds) and Level 3 (>500 beds) hospitals in 31 provinces/municipalities/autonomous regions (PMAs), representing eight economic zones in mainland China | Value congruence (congruence between an employee's personal values and an organization's culture) | Value congruence | Perceived fit scale [10] : 3 items, 6-point Likert scale | Correlation, hierarchical regression analysis | (+) Educational level (+) Job satisfaction (+) Nursing work environment (+) Title level  (-) Depersonalization (-) Emotional exhaustion (-) Sex (-) Turnover intention (-) Working experience (-) Workload (day) (-) Workload (night) | n/a | n/a | n/a | n/a | n/a |  | (+) Job satisfaction  (-) Depersonalization (-) Emotional exhaustion | n/a |
| (Sun et al., 2023) [76] | China | Cross-sectional | 614 nurses and their supervisors from five health sectors | Person–organization fit | Person–organization fit | Cable and DeRue [10] : 10 items, 5-point Likert scale | Structured model | n/a | n/a | n/a | n/a | n/a | n/a |  | (+) Organizational loyalty | n/a |
| (Teo et al., 2023) [77] | Australia | Longitudinal: Times 1 and 2 (six months after Time 1) | 230 nurses from Australia, between 18 and 65 years, full and part-time employment, employment in public, private, and not-for-profit hospitals | Person–organization fit | Person–organization fit | Cable and DeRue [10]: 3 items, 7-point Likert scale  *Measured at Time 1 | Correlation, multiple regression analysis | (+) Authentic leadership  (-) Workplace incivility | n/a | Variables measured after PO fit (×) Psychological well-being | n/a | n/a | n/a |  | n/a | (×) Psychological well-being |
| (Tomlinson, 2013) [78] | United States | Cross-sectional | 54 registered nurses providing direct patient care in a small, not-for-profit healthcare system | Person–job fit | Person–job fit | Person–Job Fit Scale [32]: 4 items, 5-point Likert scale | Independent samples t-test between two clusters of motivational gifts and units | n/a | Unit Obstetrics unit > medical unit Surgical department > medical unit  (×) No differences in person−job fit scores between the two clusters of motivational gift | n/a | n/a | n/a | n/a |  | n/a | n/a |
| (Ture et al., 2022) [79] | Turkey | Cross-sectional | 408 nurses working in a university hospital operating in Eskisehir (Turkey) | Person–organization fit | Person–organization fit | Person−Organization Fit Questionnaire [28]: 4 items, 5-point Likert scale | Correlation, three-stage regression analysis | (+) Innovative behavior (+) Leader−member exchange (+) Organizational identification | n/a | n/a | (+) Leader−member exchange (+) Organizational identification | n/a | (×) Age (×) Experience |  | (+) Innovative behavior | n/a |
| (Yan et al., 2023) [15] | China | Cross-sectional | 859 nurses employed in Xinqiao Hospital | Person–organization fit | Person–organization fit | Cable and DeRue [10]: 9 items, x-point Likert scale | Correlation,  hierarchical regression analysis, and conditional process analysis | (+) External employability (+) Internal employability (+) Organizational commitment (+) Training | n/a | n/a | n/a | n/a | n/a |  | (+) Organizational commitment | n/a |
| Note: PE fit, person–environment fit; (+), positive relationship; (-), negative relationship; (×), non-significant relationship; NS fit, needs–supplies fit; DA fit, demands–abilities fit; PO fit, person–organization fi; PS fit, person–supervisor fit; PG fit, person–group fit | | | | | | | | | | | | | | | | |
